# Label-Free Electrochemical Aptasensor Based on the Vertically-Aligned Mesoporous Silica Films for Determination of Aflatoxin B1

**DOI:** 10.3390/bios13060661

**Published:** 2023-06-16

**Authors:** Tongtong Zhang, Shuai Xu, Xingyu Lin, Jiyang Liu, Kai Wang

**Affiliations:** 1 Key Laboratory of Integrated Oncology and Intelligent Medicine of Zhejiang Province, Department of Hepatobiliary and Pancreatic Surgery, Affiliated Hangzhou First People’s Hospital, Zhejiang University School of Medicine, Hangzhou 310006, China; tongtongzhang@zju.edu.cn; 2 Key Laboratory of Surface & Interface Science of Polymer Materials of Zhejiang Province, Department of Chemistry, Zhejiang Sci-Tech University, Hangzhou 310018, China; 202120104178@mails.zstu.edu.cn; 3 College of Biosystems Engineering and Food Science, Zhejiang University, Hangzhou 310058, China; xingyu@zju.edu.cn

**Keywords:** vertically aligned mesoporous silica films, electrochemical aptasensor, aflatoxin B1

## Abstract

Herein we report a highly specific electrochemical aptasenseor for AFB1 determination based on AFB1-controlled diffusion of redox probe (Ru(NH_3_)_6_^3+^) through nanochannels of AFB1-specific aptamer functionalized VMSF. A high density of silanol groups on the inner surface confers VMSF with cationic permselectivity, enabling electrostatic preconcentration of Ru(NH_3_)_6_^3+^ and producing amplified electrochemical signals. Upon the addition of AFB1, the specific interaction between the aptamer and AFB1 occurs and generates steric hindrance effect on the access of Ru(NH_3_)_6_^3+^, finally resulting in the reduced electrochemical responses and allowing the quantitative determination of AFB1. The proposed electrochemical aptasensor shows excellent detection performance in the range of 3 pg/mL to 3 μg/mL with a low detection limit of 2.3 pg/mL for AFB1 detection. Practical analysis of AFB1 in peanut and corn samples is also accomplished with satisfactory results by our fabricated electrochemical aptasensor.

## 1. Introduction

Aflatoxins (AFs) are secondary metabolites produced by Aspergillus aflatoxins and Aspergillus parasitica, which have attracted extensive attention due to their immunotoxicity to phagocytes and cell-mediated immunity [1]. As a natural pollutant, aflatoxin can easily contaminate grains (e.g., corn, peanuts, and wheat) and some fermented foods [2]. Aflatoxin B1 (AFB1) belonging to the AF family has the highest toxicity and has been certified as a first-class carcinogen by the international cancer research institute [3]. Research has suggested that AFB1 has teratogenic, hepatotoxic, carcinogenic, mutagenic, and other adverse effects on the human body [4,5]. The EU (EC, No. 1126/2007) sets the maximum residue levels (MRLs) of 2 μg kg^−1^ for AFB1 in cereals, and Chinese food hygiene standards (GB2761-2017) stipulate that the content of aflatoxin B1 in corn, peanuts, and peanut oil should not exceed 5 μg kg^−1^ [6,7]. Therefore, it is of great significance to develop effective and reliable analytical methods for the detection of trace AFB1.

Many analytical methods have been selected for AFB1 determination, such as high-performance liquid chromatography [8], thin layer chromatography [9], liquid chromatography–mass spectrometry [10], capillary electrophoresis [11], fluorimetric/colorimetric techniques [12,13], enzyme-linked immunosorbent assay (ELISA) [14], and electrochemistry [15]. Benefiting from the advantages of fast analysis speed, simple operations, high sensitivity, and portability, electrochemical sensors display great potential in varieties of analytical fields [16,17,18,19,20].

With the development of biotechnology in recent years, aptamers have been widely used in biosensors. Aptamers are single-stranded oligonucleotides isolated from combinational DNA libraries by an extraneous process called the systematic evolution of ligands by exponential enrichment (SELEX), which has a high degree of specificity for a wide range of targets such as antibiotics, toxins, peptides, and cells [21,22]. Compared with traditional recognition elements (antibodies), aptamers have several competitive advantages, such as high specific recognition ability, low cost of chemical synthesis, good thermal stability, lack of immunogenicity, and easy modification [23]. Exploitation of aptamers for designing various biosensors based on the colorimetry, electrochemistry, fluorescence, and surface-enhanced Raman offers the possibility to determine AFB1 with high specificity [24,25,26,27].

Many nanomaterials, such as CNT, graphene, metal nanoparticles, and metal oxide nanoparticles, can be coupled to aptamers to construct optical and electrochemical sensors with good performance [28], low detection limits, and wide linear analysis ranges at subpicomolar and even subfemolar levels [29,30]. Vertically-ordered mesoporous silica films (VMSFs) that possess an ultrasmall pore size (2~3 nm), ultrathin, and high porosity have been shown to exhibit excellent permeability, extraordinary permselectivity, and good anti-fouling capacity, which have emerged as interesting materials and can modify the solid electrode substrates for the construction of numerous electrochemical sensors [31,32,33,34]. Owing to the ultrasmall pore size, the VMSF hinders the ingress of large molecules while allowing small molecules to the underlying electrode surface through the vertical silica nanochannels [35,36,37,38]. Based on the above hindered effect on the large molecules, biological recognition elements (e.g., antigen [39,40,41], antibody [42], and aptamer [43]) immobilized onto the outer surface of the VMSF have been combined with the electrochemiluminescence (ECL) luminophores or electrochemical redox probes to develop various label-free ECL and electrochemical sensors. Aptamers with a small size are more beneficial to functionalize the VMSF outer surface, effectively avoiding the pore blockage problem. To the best of our knowledge, electrochemical aptasensors based on the VMSF have not been applied to the quantitative analysis of AFB1.

In this work, highly specific electrochemical aptasensor for AFB1 determination is demonstrated based on AFB1-controlled diffusion of the redox probe (Ru(NH_3_)_6_^3+^) through the nanochannels of the AFB1-specific aptamer functionalized VMSF. Immobilization of the AFB1-specific aptamer on the outer surface of the VMSF can be accomplished by covalent modification using (3-glycidyloxypropyl) trimethoxysilane as the coupling agent. Upon the introduction of AFB1, the specific interaction between the aptamer and AFB1 can produce the steric hindrance effect on the diffusion of Ru(NH_3_)_6_^3+^, resulting in the reduced electrochemical responses and finally enabling the quantitative determination of AFB1. Moreover, a high density of silanol groups on the VMSF surface makes its surface bear negative charges in specific conditions, electrostatically attracting cationic Ru(NH_3_)_6_^3+^ and giving rise to an amplified electrochemical signal. Practical analysis of AFB1 in peanut and corn kernels is also studied by our developed electrochemical aptasensor.

## 2. Materials and Methods

### 2.1. Chemicals and Materials

Hexaammineruthenium(III) chloride ([Ru(NH_3_)_6_]Cl_3_, 98.0%) and hexadecyl trimethyl ammonium bromide (CTAB, 99%) were ordered from Sigma-Aldrich (Shanghai, China). Hydrochloric acid (HCl, 99.7%) and ethanol (EtOH, ≥98.0%) were bought from Sinopharm Group Chemical Reagent Co., Ltd. (Shanghai, China). Potassium ferricyanide (K_3_[Fe(CN)_6_], ≥98%), tetrapotassium hexacyanoferrate trihydrate (K_4_[Fe(CN)_6_], ≥98%), tetraethyl orthosilicate (TEOS, ≥99.0%), lysine, sucrose, Concanavalin A (ConA), sodium nitrate (NaNO_3_, ≥98%), (3-glycidyloxypropyl) trimethoxysilane (GOPTS, ≥99.0%), and starch soluble (≥98%) were all purchased from Aladdin Chemistry Co., Ltd. (Shanghai, China). Hydroxymethyl ferrocene (FcMeOH, 98.0%) and potassium hydrogen phthalate (KHP, 99.0%) were obtained from Maclin Biochemical Technology Co., Ltd. (Shanghai, China). Ochratoxin (OTA) and zearalenone (ZEN) were purchased from Beijing Keyue Zhongkai Biotechnology Co., Ltd. (Beijing, China). Aflatoxin B1 (AFB1), aflatoxin B2 (AFB2), bovine serum albumin (BSA, 20 mg/mL), and AFB1 aptamer (HPLC purification, ≥99%) were ordered from Sangon Biotech (Shanghai, China). The sequence of the AFB1 aptamer modified with amino groups is as follows: 5′-GTT GGG CAC GTG TTG TCT CTC TGT GTC TCG TGC CCT TCG CTA GGC CCA CA-NH_2_-3′. Peanuts and maize were bought from supermarkets. Indium tin oxide (ITO) coated glasses were obtained from Zhuhai Kaivo Electronic Components (Zhuhai, China). Prior to use, the organic residues were removed by soaking the ITO electrodes into a NaOH aqueous solution (1 M) for 12 h. Then the ITO electrodes were cleaned using ultrasonic washing with acetone, ethanol, and deionized water, in that order. Phosphate-buffered saline (PBS) was made up of Na_2_HPO_4_ and NaH_2_PO_4_•2H_2_O, and its pH was adjusted to 7.4 by HCl (0.1 M) or NaOH (0.1 M) solution. All aqueous solutions were prepared using deionized water (18.2 MΩ·cm, 25 °C), and the various chemicals used in the process were of analytical reagent grade.

### 2.2. Measurements and Instrumentations

All electrochemical experiments were carried out on an Autolab electrochemical workstation (Metrohm, Herisau, Switzerland). A traditional three-electrode system was used for electrochemical measurements. In simple terms, an Ag/AgCl (in saturated KCl) electrode was used as the reference electrode, a platinum wire electrode was used as the counter electrode, and bare ITO or modified ITO was used as the working electrode. The scan rate for cyclic voltammetry (CV) in the electrochemical tests was 50 mV/s, and the step potential, modulation amplitude, pulse width, and pulse period of differential pulse voltammetry (DPV) were 0.005 V, 0.025 V, 0.05 s, and 0.5 s, respectively. The surface morphology and structure of the VMSF were characterized using thermal field emission scanning electron microscopy (SEM, VITRA55, Tokyo, Japan) and transmission electron microscopy (TEM, JEM2100, Tokyo, Japan). For SEM and TEM testing, the actual operating voltages were 5 kV and 100 kV, respectively. An X-ray photoelectron spectroscopy (XPS) PHI5300 spectrometer (PE Ltd., Cincinnati, OH, USA) with Mg Kα ray source excitation (250 W and 14 kV) was used to characterize the modification of the aptamer.

### 2.3. Preparation of VMSF/ITO Electrode

The VMSF was grown on an ITO glass by the Stöber solution growth method using TEOS as the precursor and CTAB surfactant micelles (SMs) as the template [44]. In a nutshell, 0.44 mM CTAB was added to 100 mL of mixed solution of water and ethanol (7:3 v:v) under stirring until completely dissolved, and then 80 μL TEOS and 25 μL NH_3_•H_2_O (10% *w*/*w*) were added while maintaining the stirring state. Cleaned ITO substrates were immersed into the precursor solution and grown at 60 °C for 24 h. To obtain the stable VMSF on the electrode, the resultant modified electrode was washed with deionized water, dried in an oven at 80 °C, and aged overnight. Thus, an SM-templated VMSF was attached to the ITO surface, designated as the SM@VMSF/ITO electrode. Treatment with 0.1 M HCl/ethanol solution under stirring for 5 min might be used to remove SMs from the nanochannels of the VMSF.

### 2.4. Fabrication of Label-Free Aptamer Sensor

The AFB1-specific aptamer can be modified on the outer surface of the VMSF instead of the inside nanochannels by using GOPTS as the coupling agent. To avoid the plugging issue of silica nanochannels in the GOPTS modification process, SMs first remained inside silica nanochannels and were removed after GOPTS modification. In simple terms, SM@VMSF/ITO electrodes were immersed into 25 mL ethanol solution containing 13 μL GOPTS for 60 min before being washed with ultrapure water. After removing SMs from the silica nanochannels, GOPTS-modified VMSF surface was obtained, designated as O-VMSF/ITO. The epoxy groups of GOPTS provide the functional sites for further covalent modification of the AFB1-specific aptamer. About 50 μL of the AFB1 aptamer solution (0.5 μM) was drop-coated on the O-VMSF/ITO electrode surface and incubated for 90 min at 4 °C. After being rinsed with PBS (0.01 M, pH 7.4) to remove the unbound aptamer and incubated with BSA solution (1%, *w*/*w*) for 30 min to block the nonspecific binding sites, Apt/O-VMSF/ITO and BSA/Apt/O-VMSF/ITO electrodes were obtained, respectively.

### 2.5. Electrochemical Detection of AFB1

For the detection of AFB1: First, 50 μL of AFB1 with various concentrations was drop-coated on the BSA/Apt/O-VMSF/ITO aptamer sensor, incubated at 37 °C for 90 min, and then the unbound AFB1 was rinsed off with PBS (0.01 M, pH 7.4). Finally, electrochemical tests were performed in PBS (0.01 M, pH 7.4) containing 10 μM Ru(NH_3_)_6_^3+^, and the DPV signal of the electrode was recorded. For the actual sample analysis, 3 g moldy peanut or maize samples were soaked in 10 mL water: methanol (3:7 v:v) solution for 12 h, centrifuged for 10 min at 6000 rpm, and the supernatant was diluted 10 times with PBS (0.01 M, pH 7.4).

## 3. Results and Discussion

### 3.1. Electrochemical Sensing Strategy for AFB1 Determination

**Figure 1:**
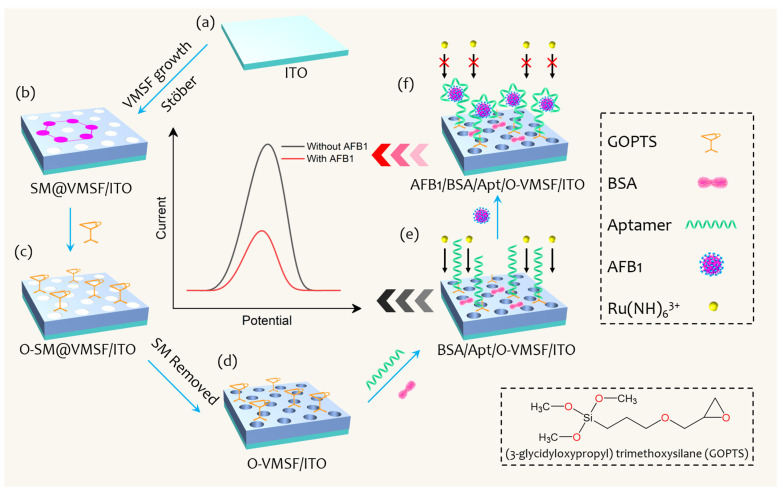
Illustration of preparation process of BSA/Apt/O-VMSF/ITO electrochemical aptasensor and AFB1 assay. (**a**) Bare ITO electrode. (**b**) Binary assembly consisting of SM and VMSF on the ITO glass. (**c**) GOPTS bearing epoxy groups functionalized VMSF/ITO. (**d**) Removal of SM. (**e**) Modification of AFB1-specific aptamer and blockage of non-specific binding sites using BSA. (**f**) Detection of AFB1 with the help of Ru(NH_3_)_6_^3+^ in the bulk solution.

**Figure 2:**
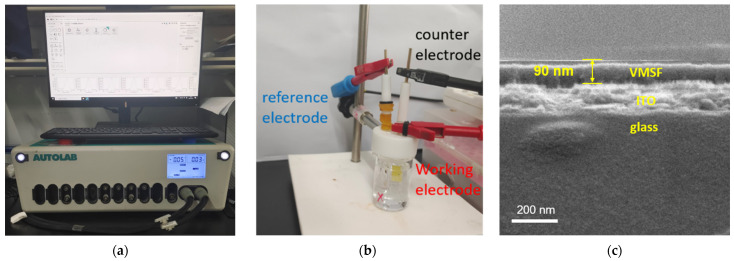

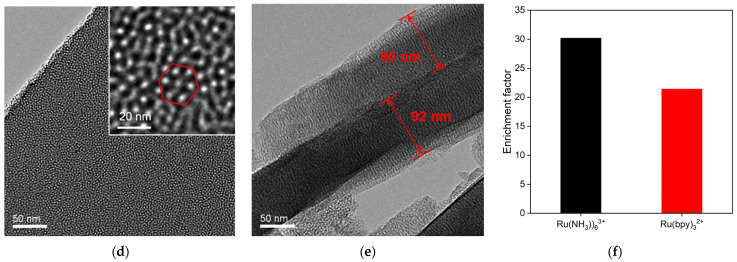
Photographs of Autolab electrochemical workstation (**a**) and detection device built for AFB1 measurement. (**b**) Cross-sectional view SEM image, (**c**) top-view, (**d**) cross-sectional view, and (**e**) TEM images of VMSF. (**f**) Enrichment factor (ratio of anodic peak currents at the VMSF/ITO and bare ITO) of anodic peak current obtained from the VMSF/ITO electrode relative to that of bare ITO in these two probe solutions.

As shown in Figure 1, the construction of an electrochemical aptasensor involves the growth of the VMSF and AFB1-specific aptamer modification. The VMSF is attached to the ITO electrode using the Stöber solution growth method. The as-prepared electrode remains the SM inside the silica nanochannels, which is directly used to the further modification with GOPTS. In this case, GOPTS with epoxy groups can be grafted on the outer surface of the VMSF instead of inner nanochannels, avoiding the plugging problem. Then the SM is removed from the silica nanochannels to achieve the epoxy group functionalized VMSF (O-VMSF/ITO) with accessible nanochannels. Thanks to the covalent reaction between the epoxy groups of GOPTS and the amino groups of the aptamer, the AFB1-specfic aptamer can be fixed on the outer surface of the VMSF. Finally, BSA is employed to block the non-specific binding sites to form an electrochemical aptasensor, designated as the BSA/Apt/O-VMSF/ITO electrode, and the silica inner walls of the VMSF bearing negative charges provide electrostatic preconcentration ability toward the electrochemical redox probe (Ru(NH_3_)_6_^3+^). When the target AFB1 is present, AFB1 specifically interacts with its aptamer to form a complex onto the BSA/Apt/O-VMSF/ITO electrode, resulting in the hindered access of Ru(NH_3_)_6_^3+^ and achieving the reduced current signal. On the basis of the above mechanism, reduced current signals at the BSA/Apt/O-VMSF/ITO aptasensor have positive correlation with AFB1 concentration, which can be recorded by electrochemical workstation using a three-electrode system (Figure 2a,b) and realize the quantitative determination of AFB1. Note that the VMSF carrying negatively charged surface in the experimental condition is capable of preconcentrating cationic Ru(NH_3_)_6_^3+^ and generating the amplified electrochemical current signal, effectively promoting the detection performance.

### 3.2. Characterizations of VMSF/ITO and Constructed Electrochemical Aptasensor

In order to understand the morphology and properties of the prepared VMSF, electron microscopes and electrochemical methods were employed. As shown from the cross-sectional SEM image in Figure 2c, the VMSF with homogeneous thickness of ~90 nm is observed on the top of the ITO coated glass. Top-view TEM images show that the nanopores of the VMSF are closely oriented in a hexagonal arrangement, and their diameter is about 2~3 nm (Figure 2d). The cross-sectional TEM image also reveals the parallel nanochannels of about 90 nm in thickness (Figure 2e). Then, three electrochemical redox probes with different characteristics, namely Fe(CN)_6_^3−^ (electronegativity), Ru(NH_3_)_6_^3+^ (electronegativity), and FcMeOH (neutrality), were selected to examine the integrity and charge permselectivity of the VMSF by using the cyclic voltammetry (CV) method. As shown in Figure S1 in Supplementary Material, the templated SM filled in the vertical silica nanochannels prohibits the access of charged probes (Fe(CN)_6_^3−^ and Ru(NH_3_)_6_^3+^) to reach the underlying ITO electrode surface, eventually blocking their electron exchange on the electrode surface. However, the ingress of neutral FcMeOH into the silica nanochannels is unhindered, indicating that silica nanochannels attached to the ITO surface are fully filled with SMs and further confirming the intact VMSF on the ITO surface. After SM exclusion, an apparent charge permselectivity is displayed at the VSMF/ITO electrode compared with that of the bare ITO electrode, electrostatically attracting Ru(NH_3_)_6_^3+^ and repelling Fe(CN)_6_^3−^, which is attributed to the deprotonation of numerous silanol groups on the inner surface at pH 7.4 (the reported pK_a_ of silanol groups on the silica nanochannel surface is about 3) [45]. On the basis of the above electrostatic effect, an amplified electrochemical signal is generated for Ru(NH_3_)_6_^3+^ and a depleted signal for Fe(CN)_6_^3−^ at the VMSF/ITO electrode, in comparison with that of the bare ITO electrode. Figure 2f and Figure S2 compare the electrostatic enrichment capacity of VMSF/ITO toward Ru(NH_3_)_6_^3+^ and [Ru(bpy)_3_]^2+^. As seen in comparison with the bare ITO electrode, VMSF/ITO displays the amplified electrochemical signals for both [Ru(bpy)_3_]^2+^ and Ru(NH_3_)_6_^3+^ with positive charge, which is attributed to the deprotonation of silanol groups on the inner surface. Furthermore, VMSF/ITO shows a higher enrichment factor for Ru(NH_3_)_6_^3+^ compared with [Ru(bpy)_3_]^2+^. Therefore, Ru(NH_3_)_6_^3+^ was chosen as the electrochemical probe in this study. In addition, as the half-wave width of the DPV curve represents the electrochemical behavior and reaction rate of the electroactive molecules, the DPV curves of bare ITO and VMSF/ITO electrodes in PBS solution containing 100 μM Ru(NH_3_)_6_^3+^ are recorded in Figure S3. As shown, the half-wave width of the DPV curve obtained at the VMSF/ITO electrode is 20 mV lower than that of bare ITO, indicating the faster electrochemical reaction rate of Ru(NH_3_)_6_^3+^ at the VMSF/ITO electrode and further confirming the electrostatic enrichment capacity of the VMSF bearing negative charge.

**Figure 3:**
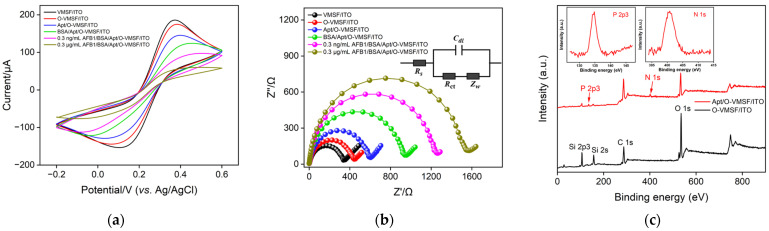
CV curves (**a**) and EIS plots (**b**) of VMSF/ITO, O-VMSF/ITO, Apt/O-VMSF/ITO, BSA/Apt/O-VMSF/ITO, and AFB1/BSA/Apt/O-VMSF/ITO electrodes obtained in 0.1 M KCl containing 2.5 mM Fe(CN)_6_^3−/4−^. The inset in (**b**) is an equivalent circuit, including solution resistance (*R*_s_), two-layer capacitance (*C*_dl_), Warburg impedance (*Z*_w_), and *R*_ct_. (**c**) XPS profiles of O-VMSF/ITO and Apt/O-VMSF/ITO electrodes. Insets show the diffraction peaks of the N and P elements.

CV, electrochemical impedance (EIS), and XPS were then used to demonstrate the construction procedure of our electrochemical aptasensor, and the corresponding results are shown in Figure 3. It can be found from Figure 3a,b that the functionalization of epoxy groups on the outer surface of the VMSF gives rise to the slight decrease in redox current values and minor increased transparent charge transfer resistance (*R*_ct_), suggesting GOPTS grafted on the outer surface of the VMSF indeed does not produce the plugging problem. AFB1-spefic aptamers and BSA are consecutively modified on the VMSF surface to obtain the BSA/Apt/O-VMSF/ITO electrode, generating decreased redox current values and enhanced *R*_ct_, which is ascribed to the steric hindrance effect on the transport of the redox probe. When 0.3 ng/mL or 0.3 μg/mL AFB1 is introduced, specific interaction occurs and more distinct hindrance effect causes a significant decrease in redox currents and increase in *R*_ct_, confirming that the proposed BSA/Apt/O-VMSF/ITO sensor for AFB1 is feasible and effective. Moreover, as shown in the XPS results in Figure 3c, the reduction of O and Si elements and the appearance of N and P elements are observed at the Apt/O-VMSF/ITO electrode, further indicating the successful attachment of aptamers to the O-VMSF/ITO electrode.

### 3.3. Optimum Conditions for the Detection of AFB1

**Figure 4:**
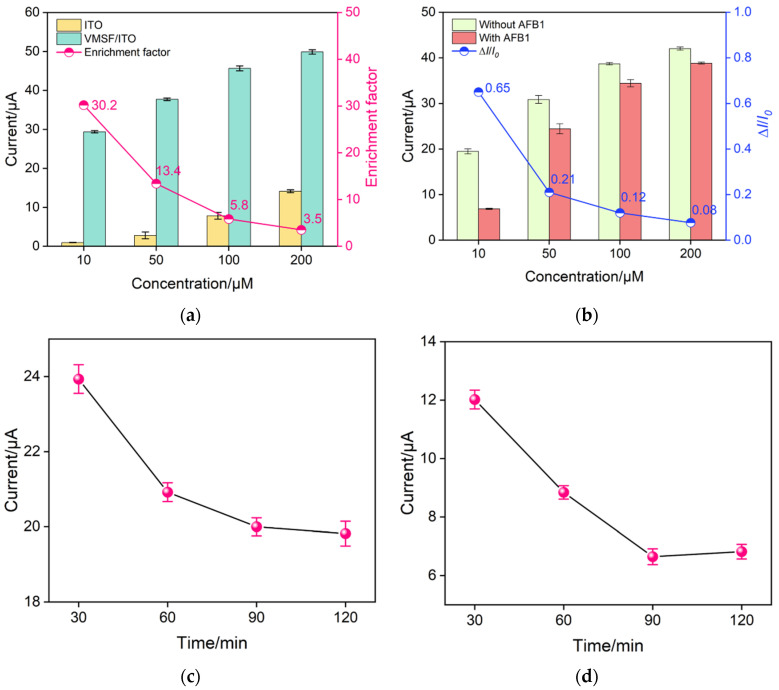
(**a**) Anodic peak currents of Ru(NH_3_)_6_^3+^ with different concentrations at the bare ITO and VMSF/ITO electrodes (bar graphs) and enrichment factors for Ru(NH_3_)_6_^3+^ by VMSF/ITO (point plot). (**b**) Anodic peak currents (bar graphs) and current variation ratio (point plot) obtained at the BSA/Apt/O-VMSF/ITO electrodes before and after incubation with 300 ng/mL AFB1 in PBS solution containing different concentrations of Ru(NH_3_)_6_^3+^. Optimization of incubation time for aptamer (**c**) and AFB1 (**d**). Error bars represent the standard deviation of the results measured in three parallel experiments.

As cationic Ru(NH_3_)_6_^3+^ can be electrostatically preconcentrated by the VMSF to produce strong electrochemical signals, the concentration of Ru(NH_3_)_6_^3+^ was first optimized. Figure 4a records the anodic peak currents of Ru(NH_3_)_6_^3+^ with various concentrations at the bare ITO and VMSF/ITO electrodes. To quantitatively access the electrostatic preconcentration ability of the VMSF, enrichment factor is defined as the ratio of anodic peak currents obtained at the VMSF/ITO and bare ITO electrode. As seen, the enrichment factor gradually declines with the increasing Ru(NH_3_)_6_^3+^ concentration, which is due to the decreased thickness of the electric double layer inside the ultrasmall nanochannels. Figure 4b shows the electrochemical responses of the BSA/Apt/O-VMSF/ITO aptasensor in the absence (*I*_0_) and presence (*I*) of AFB1. The current variation ratio (Δ*I*/*I*_0_, Δ*I* = *I*_0_ − *I*) becomes more and more smaller with the increasing Ru(NH_3_)_6_^3+^ concentration, which is attributed to the large concentration gradient near the electrode interface at the high concentration of Ru(NH_3_)_6_^3+^. Therefore, 10 μM is selected as the appropriate concentration for AFB1 determination. In addition, the incubation times for the AFB1-specific aptamer and AFB1 are noteworthy conditions. Appropriate incubation time can ensure the best signal while shortening the preparation time of sensors. As shown in Figure 4c and Figure S4, the required incubation time for an aptamer increases with the increasing aptamer concentration. Considering the cost and time, a 0.5 μM aptamer with 90 min incubation time is chosen as the optimal experimental condition in this study. Under the optimal Ru(NH_3_)_6_^3+^ concentration (10 μM), the optimal incubation time for AFB1 is 90 min (Figure 4d).

### 3.4. Sensitive Detection of AFB1 Using BSA/Apt/O-VMSF/ITO

**Figure 5:**
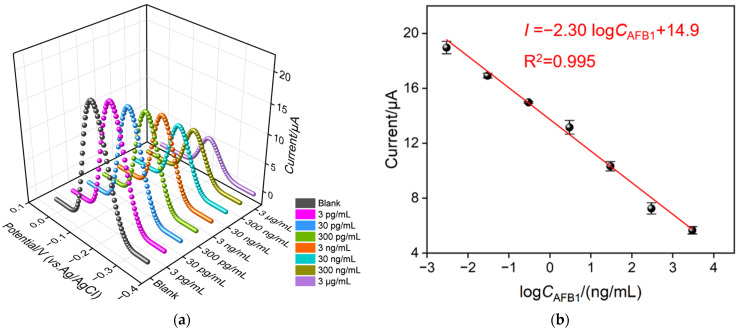
(**a**) DPV curves of BSA/Apt/O-VMSF/ITO aptasensor in presence of different concentrations of AFB1 (3 pg/mL–3 μg/mL). (**b**) Corresponding calibration curve.

To examine the electrochemical detection performance, the BSA/Apt/O-VMSF/ITO electrode was incubated with different concentrations of AFB1 and tested in PBS solution containing 10 μM Ru(NH_3_)_6_^3+^ (0.01 M pH 7.4) under optimal experimental conditions using the DPV technique. As displayed in Figure 5a,b and Table S1, the anodic peak current signal is linearly proportional to the logarithmic value of the AFB1 concentration in the range of 3 pg/mL–3 μg/mL, yielding a fitting linear equation of *I* (μA) = 2.30 log*C*_AFB1_ (ng/mL) + 14.9 (R^2^ = 0.995) and the limit of detection (LOD) of 2.3 pg/mL (S/N = 3). The LOD was obtained according to the calculation method prescribed by the international union of pure and applied chemistry (IUPAC), which is expressed as the concentration (*C*_L_) and derived from the minimum measurement (*X*_L_). The value of *X*_L_ is given by the equation *X*_L_ = x_b1_ + *k*s_b1_, where x_b1_ and s_b1_ are the mean and standard deviation of blank measurements, respectively, and the *k* value is generally 3, which in practical sense usually corresponds to a confidence level of about 90%. The LOD was derived by bringing the minimum measured value (*X*_L_) into the calibration curve equation obtained by a given analytical procedure [46]. Table 1 compares the detection performance of our BSA/Apt/O-VMSF/ITO aptasensor with previously published electrochemical aptamer sensors. Our proposed BSA/Apt/O-VMSF/ITO aptasensor displays several advantages of wide detection range and low LOD without complex labeling and modification process. 

### 3.5. Selectivity, Reproducibility, and Stability of Aptamer Sensors

**Figure 6:**
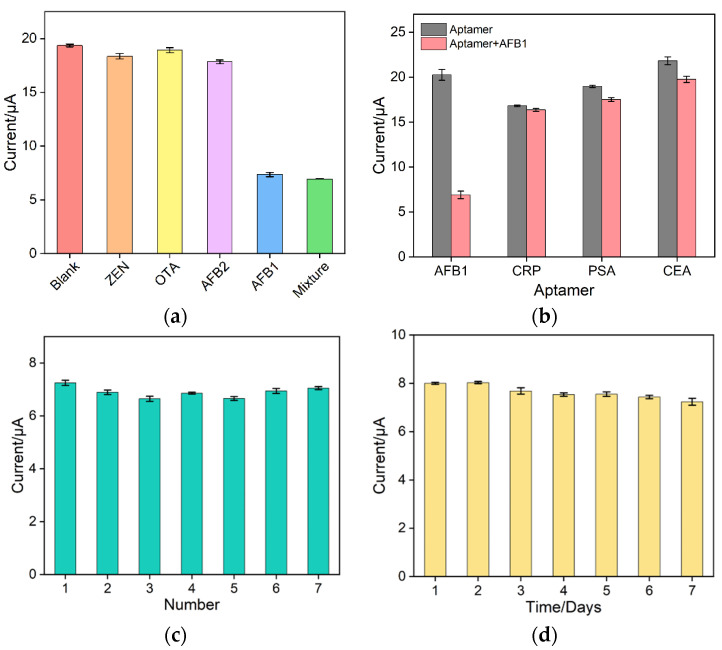
(**a**) Selectivity of aptamer sensor. Concentrations of AFB1 and other interfering species were 300 ng/mL and 3 μg/mL, respectively. Error bars represent test results from three experiments. (**b**) DPV signals of three different aptamers (corresponding to CRP, PSA, and CEA) before and after incubation with 300 ng/mL AFB1 in PBS solution containing 10 μM Ru(NH_3_)_6_^3+^. (**c**) Reproducibility of the aptamer sensor. Current signals were selected from seven electrodes in different batches after incubation with 300 ng/mL of AFB1. Error bars represent the standard deviations of three measurements for each electrode. (**d**) Stability of the BSA/Apt/O-VMSF/ITO sensor. Seven electrodes prepared in the same batch were placed at 4 °C for different days to detect the current signal of 300 ng/mL AFB1. Error bars represent the standard deviations of the results measured in three parallel experiments.

Some indicators including selectivity, reproducibility, and stability are important to evaluate the performance of the BSA/Apt/O-VMSF/ITO electrochemical aptasensor, and these results are shown in Figure 6. Three possible interfering substances, including zearalenone (ZEN), ocheratoxin A (OTA), and AFB2, were selected to interact with the BSA/Apt/O-VMSF/ITO aptasensor under the same conditions and then tested in PBS solution containing 10 μM Ru(NH_3_)_6_^3+^ (0.01 M pH 7.4) using the DPV method. As presented in Figure 6a, the current signals of starch, OTA, and ZEN are similar to that of blank samples. Only when the target AFB1 is present and even mixed with other interfering species can produce strong signal changes, indicating the high specificity of the prepared aptasensor. Three different aptamers (CRP-specific aptamer, PSA-specific aptamer, and CEA-specific aptamer) were used to prepare an electrochemical aptasensor using the same procedure and have been incubated with AFB1. As seen in Figure 6b, only the electrochemical aptasensor prepared by the AFB1-specific aptamer has effective response to AFB1, indicating the good specificity of the aptamer. The fabricated BSA/Apt/O-VMSF/ITO aptasensor cannot be regenerated after use, and reproducibility was then studied. Seven identical BSA/Apt/O-VMSF/ITO electrodes were prepared in parallel and incubated with 300 ng/mL AFB1; the obtained current values were similar with relative standard deviation (RSD) of 3.1%, suggesting the good reproducibility of our fabricated sensor (Figure 6c). In addition, when the electrode is stored at 4 °C, 90% of the initial measured signal is remained after 7 days, confirming the good long-term stability (Figure 6d).

### 3.6. Detection of AFBI in Real Samples

**Figure 7:**
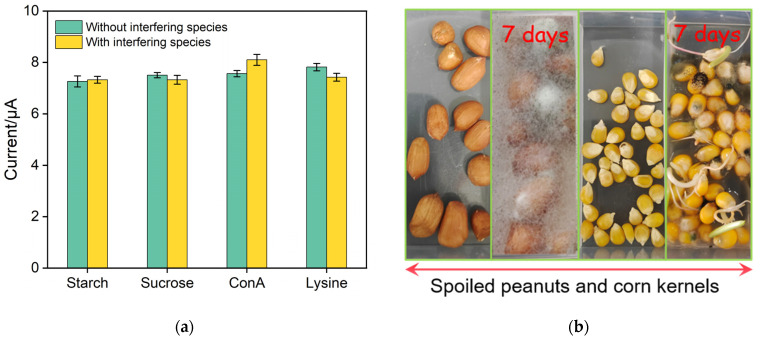
(**a**) DPV signal values of BSA/Apt/O-VMSF/ITO electrodes after incubation with 300 ng/mL AFB1 with or without 1 mg/mL of different interfering species. Error bars represent measurement errors from three experiments. (**b**) Photographs of peanut and corn kernels before and after culturing in humid and sultry environment for 7 days.

In general, AFB1 exists in mildewed grains (corn, peanuts, wheat, etc.) and will threaten human health if consumed. Four fouling agents potentially existed in grains: starch, sucrose, ConA, and lysine were employed to examine the anti-fouling property of the fabricated electrochemical aptasensor. As shown in Figure 7a, no significant signal variation is observed in the presence of both AFB1 and fouling agents. Peanuts and corn cultured for 7 days in humid and sultry environment shown in Figure 7b were selected as test objects to study the reliability and accuracy of the BSA/Apt/O-VMSF/ITO aptasensor in practical application using recovery assay. As shown in Table 2, recoveries in the range of 97.5–108% and RSD (<3%) are obtained, showing the satisfactory performance in real samples. Moreover, Ru(NH_3_)_6_^3+^, Ca^+^, K^+^, and Na^+^ were added to the extracted corn sample and tested by the BSA/Apt/O-VMSF/ITO aptasensor before and after the rinsing procedure. As shown in Figure S5, small molecules that coexisted in the corn sample cannot affect the detection of AFB1.

## 4. Conclusions

In summary, we proposed an electrochemical aptasensor for the sensitive detection of AFB1 by combining the high specificity of the AFB1-specific aptamer and signal amplification effect afforded by the VMSF. The VMSF carrying negative charges enables the electrostatic preconcentration of the cationic redox probe (Ru(NH_3_)_6_^3+^) and gives rise to strong redox signals, effectively sparing the cost of reagents while significantly enhancing the detection sensitivity. Target AFB1 can specifically interact with the AFB1-specific aptamer covalently immobilized on the outer surface of the VMSF, ultimately leading to the steric hindrance effect on the transport of Ru(NH_3_)_6_^3+^ and realizing the quantitative determination of AFB1 with a linear range of 3 pg/mL–3 μg/mL and a low LOD of 2.3 pg/mL. Moreover, satisfactory recoveries between 97.5% and 105% and relatively low RSD for the analysis of real samples (peanut and corn) can be achieved at the developed BSA/Apt/O-VMSF/ITO aptasensor. Our designed aptasensor provides a universal platform that can be applied to detect various mycotoxins by conveniently tailoring the kind of the aptamer.

## Figures and Tables

**Table 1:** Comparison of detection performance of different electrochemical aptasensors for detection of AFB1.

Electrode	Method	Tag	Number of Interfering Species	Long-Term Stability (Day)	Linear Range (pg/mL)	LOD (pg/mL)	Ref.
Apt/p-GNs-PS-COOH/GCE	EIS	Free	1	30	10–100	2	[47]
Fc-Apt/MB-cDNA/AuNFs/ITO	DPV	Fc-Apt	12	15	0.1–1 × 10^3^	0.032	[48]
Apt/AuNPs/GCE	DPV	Free	3	15	10–10^5^	1.8	[49]
Apt/Fe_3_O_4_@Au MBs/SPCE	EIS	Free	3	–	20–5 × 10^4^	15	[50]
DNA1-AuNPs-Thi/MoS_2_-rGO/AuE	DPV	Free	5	14	1–1 × 10^5^	0.3	[51]
Fc-Apt/MCH/AQ-ssDNA/AuE	ACV	Fc-Apt	5	7	10–3 × 10^3^	4.3	[52]
BSA/Apt/O-VMSF/ITO	DPV	Free	4	7	3–3 × 10^6^	2.3	This work

Apt: aptamer; p-GNs: N-doped graphene nanosheets; PS-COOH: carboxyl-functionalized polystyrene; Fc-Apt: the ferrocene-labeled AFB1 aptamer; MB-cDNA: the methylene blue labeled complementary DNA of aptamer; AuNFs: Au nanoflowers; GCE: glassy carbon electrodes; Fe_3_O_4_@Au MBs: core/shell Fe_3_O_4_@Au magnetic beads; SPCE: screen-printed carbon electrodes; AuNPs: gold nanobipyramids; Thi: thionin; MoS_2_-rGO: three-dimensional molybdenum disulfide-reduced graphene oxide; MCH: 6-mercapto-1-hexanol; AQ-ssDNA: anthraquinone-2-carboxylic acid-labeled complementary DNA; AuE: gold electrode; ACV, alternating current voltammetry.

**Table 2:** Determination of AFB1 in peanut and corn samples.

Sample ^1^	Spiked (ng/mL)	Found (ng/mL)	RSD (%)	Recovery (%)
Peanut	0.100	0.0975	1.3	97.5
	10.0	10.8	3.1	108
	100	105.4	0.9	105
Maize	0.100	0.0981	0.7	98.1
	10.0	10.0	1.0	103
	100	104	2.5	104

^1^ The sample was diluted 10 times with PBS electrolyte. The concentration of AFB is the diluted concentration.

## Data Availability

The data presented in this study are available on request from the corresponding author.

## Supplementary Materials

The following supporting information can be downloaded at: https://www.mdpi.com/article/10.3390/bios13060661/s1, Figure S1: Electrochemical characterization of VMSF/ITO; Figure S2: Electrochemical behaviors of Ru(NH_3_)_6_^3+^ and Ru(bpy)_3_^2+^ at the bare ITO and VMSF/ITO electrodes; Figure S3: Difference in half-wave width of DPV between bare ITO and VMSF/ITO; Figure S4: Optimization of aptamer concentration; Figure S5: Effect of rinsing procedure on the aptasensor; Table S1: Anodic peak currents and their standard deviations corresponding to different concentrations.

## References

[1] Pérez-Fernández B., de la Escosura-Muñiz A. (2022). Electrochemical biosensors based on nanomaterials for aflatoxins detection: A review (2015–2021). Anal. Chim. Acta.

[2] Mutiga S.K., Hoffmann V., Harvey J.W., Milgroom M.G., Nelson R.J. (2015). Assessment of aflatoxin and fumonisin contamination of maize in western kenya. Phytopathology.

[3] Gacem M.A., Ould El Hadj-Khelil A. (2016). Toxicology, biosynthesis, bio-control of aflatoxin and new methods of detection. Asian Pac. J. Trop. Biomed..

[4] Li S., Muhammad I., Yu H., Sun X., Zhang X. (2019). Detection of Aflatoxin adducts as potential markers and the role of curcumin in alleviating AFB1-induced liver damage in chickens. Ecotoxicol. Environ. Saf..

[5] Li H., Li S., Yang H., Wang Y., Wang J., Zheng N. (2019). l-Proline alleviates kidney injury caused by AFB1 and AFM1 through regulating excessive apoptosis of kidney cells. Toxins.

[6] Liu R., Li W., Cai T., Deng Y., Ding Z., Liu Y., Zhu X., Wang X., Liu J., Liang B. (2018). TiO(2) nanolayer-enhanced fluorescence for simultaneous multiplex mycotoxin detection by aptamer microarrays on a porous silicon surface. ACS Appl. Mater. Interfaces.

[7] Li Y., Liu D., Zhu C., Shen X., Liu Y., You T. (2020). Sensitivity programmable ratiometric electrochemical aptasensor based on signal engineering for the detection of aflatoxin B1 in peanut. J. Hazard. Mater..

[8] Turksoy S., Kabak B. (2020). Determination of aflatoxins and ochratoxin A in wheat from different regions of Turkey by HPLC with fluorescence detection. Acta Aliment..

[9] Priyanka S.R., Venkataramana M., Kumar G.P., Rao V.K., Murali H.C.S., Batra H.V. (2014). Occurrence and molecular detection of toxigenic Aspergillus species in food grain samples from India. J. Sci. Food Agric..

[10] Capriotti A.L., Cavaliere C., Foglia P., Samperi R., Stampachiacchiere S., Ventura S., Laganà A. (2014). Multiclass analysis of mycotoxins in biscuits by high performance liquid chromatography–tandem mass spectrometry. Comparison of different extraction procedures. J. Chromatogr. A.

[11] Wang A., Liu J., Yang J., Yang L. (2023). Aptamer affinity-based microextraction in-line coupled to capillary electrophoresis mass spectrometry using a porous layer/nanoparticle -modified open tubular column. Anal. Chim. Acta.

[12] Zhu W., Li L., Zhou Z., Yang X., Hao N., Guo Y., Wang K. (2020). A colorimetric biosensor for simultaneous ochratoxin A and aflatoxins B1 detection in agricultural products. Food Chem..

[13] Dou X., Wu G., Ding Z., Xie J. (2023). Construction of a nanoscale metal-organic framework aptasensor for fluorescence ratiometric sensing of AFB1 in real samples. Food Chem..

[14] Dai H., Huang Z., Liu X., Bi J., Shu Z., Xiao A., Wang J. (2022). Colorimetric ELISA based on urease catalysis curcumin as a ratiometric indicator for the sensitive determination of aflatoxin B1 in grain products. Talanta.

[15] Feng Z., Gao N., Liu J., Li H. (2020). Boron-doped diamond electrochemical aptasensors for trace aflatoxin B1 detection. Anal. Chim. Acta.

[16] Zheng W., Su R., Yu G., Liu L., Yan F. (2022). Highly sensitive electrochemical detection of paraquat in environmental water samples using a vertically ordered mesoporous silica film and a nanocarbon composite. Nanomaterials.

[17] Zhang M., Zou Y., Zhou X., Yan F., Ding Z. (2022). Vertically-ordered mesoporous silica films for electrochemical detection of Hg(II) ion in pharmaceuticals and soil samples. Front. Chem..

[18] Zou Y., Zhou X., Xie L., Tang H., Yan F. (2022). Vertically-ordered mesoporous silica films grown on boron nitride-graphene composite modified electrodes for rapid and sensitive detection of carbendazim in real samples. Front. Chem..

[19] Zhu X., Xuan L., Gong J., Liu J., Wang X., Xi F., Chen J. (2022). Three-dimensional macroscopic graphene supported vertically-ordered mesoporous silica-nanochannel film for direct and ultrasensitive detection of uric acid in serum. Talanta.

[20] Yan L., Zhang C., Xi F. (2022). Disposable amperometric label-free immunosensor on chitosan–graphene-modified patterned ITO electrodes for prostate specific antigen. Molecules.

[21] Khan S., Hussain A., Fahimi H., Aliakbari F., Haj Bloukh S., Edis Z., Mahdi Nejadi Babadaei M., Izadi Z., Shiri Varnamkhasti B., Jahanshahi F. (2022). A review on the therapeutic applications of aptamers and aptamer-conjugated nanoparticles in cancer, inflammatory and viral diseases. Arabian J. Chem..

[22] Afrasiabi S., Pourhajibagher M., Raoofian R., Tabarzad M., Bahador A. (2020). Therapeutic applications of nucleic acid aptamers in microbial infections. J. Biomed. Sci..

[23] Hou Y., Jia B., Sheng P., Liao X., Shi L., Fang L., Zhou L., Kong W. (2022). Aptasensors for mycotoxins in foods: Recent advances and future trends. Compr. Rev. Food Sci. Food Saf..

[24] Liu C., Wu T., Zeng W., Liu J., Hu B., Wu L. (2022). Dual-signal electrochemical aptasensor involving hybridization chain reaction amplification for aflatoxin B1 detection. Sens. Actuator B-Chem..

[25] Abnous K., Danesh N.M., Alibolandi M., Ramezani M., Sarreshtehdar Emrani A., Zolfaghari R., Taghdisi S.M. (2017). A new amplified π-shape electrochemical aptasensor for ultrasensitive detection of aflatoxin B1. Biosens. Bioelectron..

[26] Yugender Goud K., Catanante G., Hayat A., Satyanarayana M., Vengatajalabathy Gobi K., Marty J.L. (2016). Disposable and portable electrochemical aptasensor for label free detection of aflatoxin B1 in alcoholic beverages. Sens. Actuator B-Chem..

[27] Jia Y., Zhou G., Liu P., Li Z., Yu B. (2019). Recent development of aptamer sensors for the quantification of aflatoxin B1. Appl. Sci..

[28] Goud K.Y., Reddy K.K., Satyanarayana M., Kummari S., Gobi K.V. (2019). A review on recent developments in optical and electrochemical aptamer-based assays for mycotoxins using advanced nanomaterials. Microchim. Acta..

[29] Li Q., Lu Z., Tan X., Xiao X., Wang P., Wu L., Shao K., Yin W., Han H. (2017). Ultrasensitive detection of aflatoxin B1 by SERS aptasensor based on exonuclease-assisted recycling amplification. Biosens. Bioelectron..

[30] Wei M., Zhang W. (2018). Ultrasensitive aptasensor with DNA tetrahedral nanostructure for Ochratoxin A detection based on hemin/G-quadruplex catalyzed polyaniline deposition. Sens. Actuator B-Chem..

[31] Walcarius A. (2021). Electroinduced surfactant Self-assembly driven to vertical growth of oriented mesoporous films. Acc. Chem. Res..

[32] Chen H., Huang J., Zhang R., Yan F. (2022). Dual-mode electrochemiluminescence and electrochemical sensor for alpha-fetoprotein detection in human serum based on vertically ordered mesoporous silica films. Front. Chem..

[33] Huang L., Su R., Xi F. (2023). Sensitive detection of noradrenaline in human whole blood based on Au nanoparticles embedded vertically-ordered silica nanochannels modified pre-activated glassy carbon electrodes. Front. Chem..

[34] Lv N., Qiu X., Han Q., Xi F., Wang Y., Chen J. (2022). Anti-biofouling electrochemical sensor based on the binary nanocomposite of silica nanochannel array and graphene for doxorubicin detection in human serum and urine samples. Molecules.

[35] Serrano M.B., Despas C., Herzog G., Walcarius A. (2015). Mesoporous silica thin films for molecular sieving and electrode surface protection against biofouling. Electrochem. Commun..

[36] Su R., Tang H., Xi F. (2022). Sensitive electrochemical detection of p-nitrophenol by pre-activated glassy carbon electrode integrated with silica nanochannel array film. Front. Chem..

[37] Zhou H., Ding Y., Su R., Lu D., Tang H., Xi F. (2022). Silica nanochannel array film supported by β-cyclodextrin-functionalized graphene modified gold film electrode for sensitive and direct electroanalysis of acetaminophen. Front. Chem..

[38] Cui Y., Zhang S., Zhou X., Yan F., Hu W. (2023). Silica nanochannel array on co-electrodeposited graphene-carbon nanotubes 3D composite film for antifouling detection of uric acid in human serum and urine samples. Microchem. J..

[39] Huang J., Zhang T., Zheng Y., Liu J. (2023). Dual-mode sensing platform for cancer antigen 15-3 determination based on a silica nanochannel array using electrochemiluminescence and electrochemistry. Biosensors.

[40] Chen D., Luo X., Xi F. (2023). Probe-integrated electrochemical immunosensor based on electrostatic nanocage array for reagentless and sensitive detection of tumor biomarker. Front. Chem..

[41] Chang Q., Huang J., He L., Xi F. (2022). Simple immunosensor for ultrasensitive electrochemical determination of biomarker of the bone metabolism in human serum. Front. Chem..

[42] Gong J., Zhang T., Luo T., Luo X., Yan F., Tang W., Liu J. (2022). Bipolar silica nanochannel array confined electrochemiluminescence for ultrasensitive detection of SARS-CoV-2 antibody. Biosens. Bioelectron..

[43] Wu M.S., Sun X.T., Zhu M.J., Chen H.Y., Xu J.J. (2015). Mesoporous silica film-assisted amplified electrochemiluminescence for cancer cell detection. Chem. Commun..

[44] Teng Z., Zheng G., Dou Y., Li W., Mou C.Y., Zhang X., Asiri A.M., Zhao D. (2012). Highly ordered mesoporous silica films with perpendicular mesochannels by a simple stöber-solution growth approach. Angew. Angew. Chem. Int. Ed..

[45] Etienne M., Quach A., Grosso D., Nicole L., Sanchez C., Walcarius A. (2007). Molecular transport into mesostructured silica thin films:  electrochemical monitoring and comparison between *p*6 *m*, P6_3_/*Mmc*, and *Pm*3 *n* structures. Chem. Mater..

[46] Chen L., Li Y., Miao L., Pang X., Li T., Qian Y., Li H. (2021). “Lighting-up” curcumin nanoparticles triggered by pH for developing improved enzyme-linked immunosorbent assay. Biosens. Bioelectron..

[47] Lin T., Shen Y. (2020). Fabricating electrochemical aptasensors for detecting aflatoxin B1 via layer-by-layer self-assembly. J. Electroanal. Chem..

[48] Cui H., An K., Wang C., Chen Y., Jia S., Qian J., Hao N., Wei J., Wang K. (2022). A disposable ratiometric electrochemical aptasensor with exonuclease I-powered target recycling amplification for highly sensitive detection of aflatoxin B1. Sens. Actuator B-Chem..

[49] Zhong T., Li S., Li X., JiYe Y., Mo Y., Chen L., Zhang Z., Wu H., Li M., Luo Q. (2022). A label-free electrochemical aptasensor based on AuNPs-loaded zeolitic imidazolate framework-8 for sensitive determination of aflatoxin B1. Food Chem..

[50] Wang C., Qian J., An K., Ren C., Lu X., Hao N., Liu Q., Li H., Huang X., Wang K. (2018). Fabrication of magnetically assembled aptasensing device for label-free determination of aflatoxin B1 based on EIS. Biosens. Bioelectron..

[51] Yu Y., Han J., Yin J., Huang J., Liu J., Geng L., Sun X., Zhao W. (2022). Dual-target electrochemical sensor based on 3D MoS(2)-rGO and aptamer functionalized probes for simultaneous detection of mycotoxins. Front. Chem..

[52] Zhu C., Liu D., Li Y., Ma S., Wang M., You T. (2021). Hairpin DNA assisted dual-ratiometric electrochemical aptasensor with high reliability and anti-interference ability for simultaneous detection of aflatoxin B1 and ochratoxin A. Biosens. Bioelectron..

