# Supplementary material for: Label-Free Electrochemical Aptasensor Based on the Vertically-Aligned Mesoporous Silica Films for Determination of Aflatoxin B1
Source: Biosensors (Basel). 2023 Jun 16;13(6):661. doi: 10.3390/bios13060661 (PMC10296088; doi:10.3390/bios13060661)
Supplement: Supplementary file 1 [file biosensors-13-00661-s001.zip › biosensors-2387095-supplementary.pdf]

Supporting Information

Label-Free Electrochemical Aptasensor Based on the Vertically-Aligned Mesoporous Silica Films for Determination of Aflatoxin B1

Tongtong Zhang 1,†, Shuai Xu 2,†, Xingyu Lin 3, Jiyang Liu 2,* and Kai Wang 1,*

[1] Key Laboratory of Integrated Oncology and Intelligent Medicine of Zhejiang Province, Department of Hepatobiliary and Pancreatic Surgery, Affiliated Hangzhou First People's Hospital, Zhejiang University School of Medicine, Hangzhou 310006, China

[2] Key Laboratory of Surface & Interface Science of Polymer Materials of Zhejiang Province, Department of Chemistry, Zhejiang Sci-Tech University, Hangzhou 310018, China

[3] College of Biosystems Engineering and Food Science, Zhejiang University, Hangzhou 310058, China

[*] Correspondence: liujy@zstu.edu.cn (J.L.); kaiw3@zju.edu.cn (K.W.)

[†] These authors contributed equally to this work.

Table of Contents

S1. Electrochemical characterization of VMSF/ITO

S2. Electrochemical behaviors of $Ru(NH_3)_6^{3+}$ and $Ru(bpy)_3^{2+}$ at the bare ITO and VMSF/ITO electrodes

S3. Difference in half-wave width of DPV between bare ITO and VMSF/ITO

S4. Optimization of aptamer concentration

S5. Effect of rinsing procedure on the aptasensor

S6. Anodic peak currents and their standard deviations corresponding to different concentrations

S1. Electrochemical characterization of VMSF/ITO

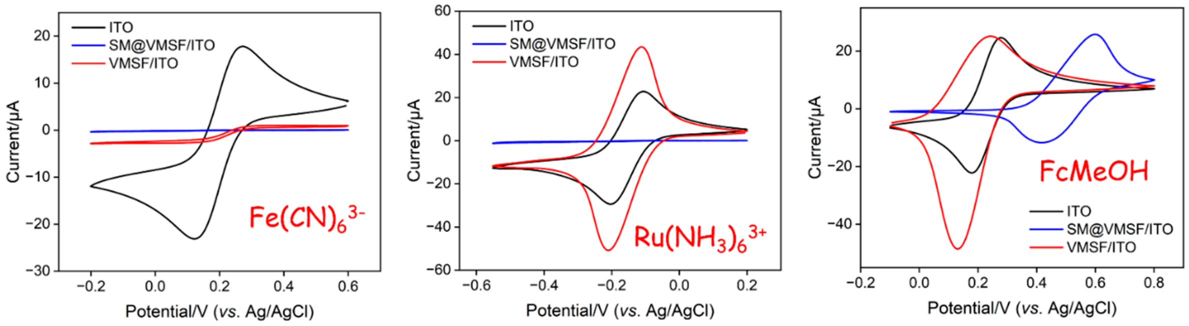

(a) (b) (c)

Figure S1 (a-c) CV curves of bare ITO, SM@VMSF/ITO and VMSF/ITO electrodes in 0.05 M KHP solution (pH = 7.4) containing 0.5 mM $K_3[Fe(CN)_6]$ (a), $[Ru(NH_3)_6]Cl_3$ (b) and FcMeOH (c), respectively.

S2. Electrochemical behaviors of $Ru(NH_3)_6^{3+}$ and $Ru(bpy)_3^{2+}$ at the bare ITO and VMSF/ITO electrodes

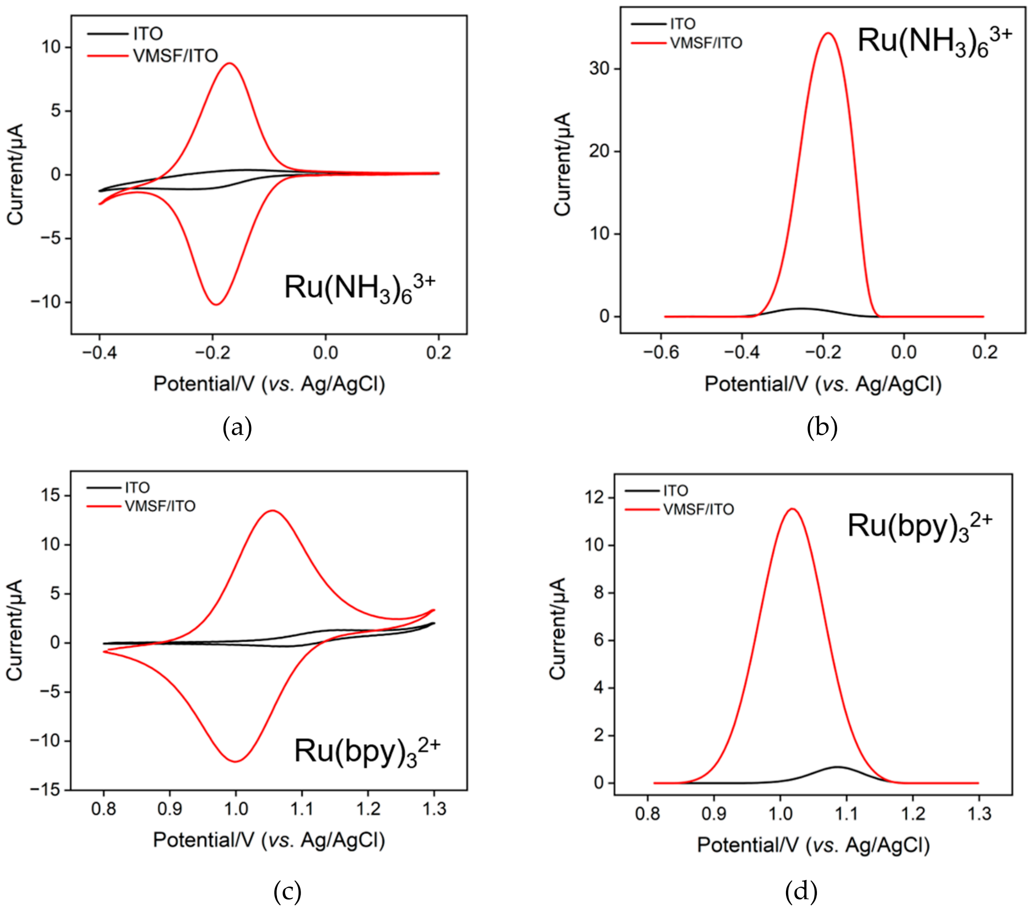

Figure S2 CV (a, c) and DPV (b, d) curves of bare ITO (black line) and VMSF/ITO (red line) in 0.01M PBS (pH=7.4) solution containing 10 μM $Ru(NH_3)_6^{3+}$ (a, b) and $Ru(bpy)_3^{2+}$ (c, d), respectively.

S3. Difference in half-wave width of DPV between bare ITO and VMSF/ITO

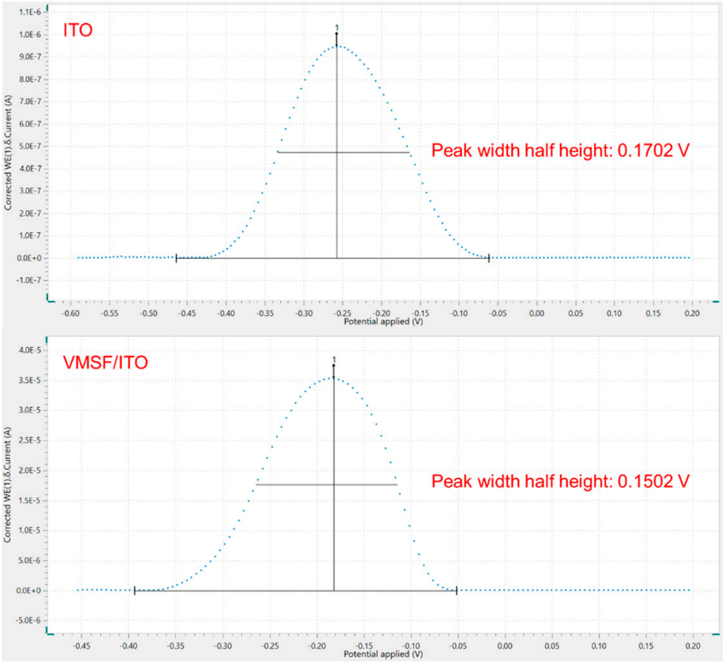

Figure S3 DPV curves of bare ITO and VMSF/ITO electrodes in PBS (0.01M pH=7.4) solution containing 10 μM $Ru(NH_3)_6^{3+}$.

S4 Optimization of aptamer concentration

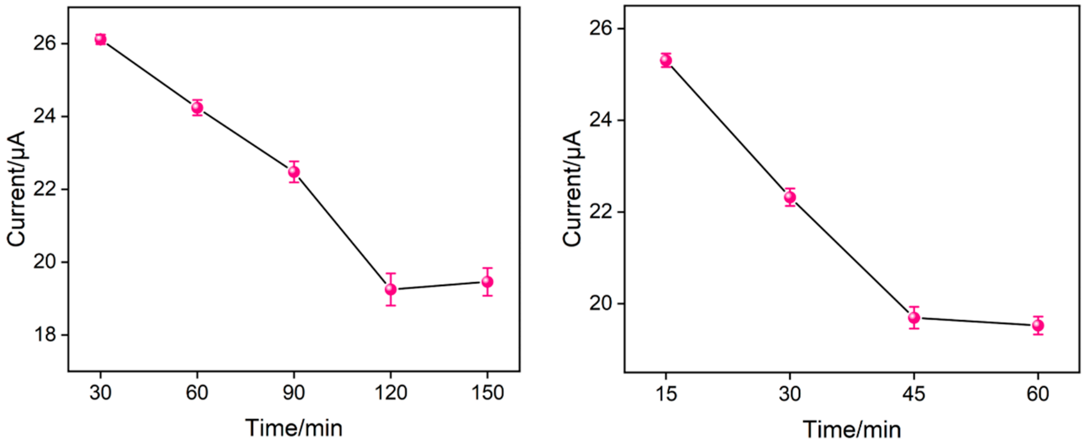

(a) (b)

Figure S4 Optimization of incubation time for aptamer with different concentrations: (a) 0.1 μM and (b) 1 μM. Error bars represent the standard deviation of three experimental measurements.

S5. Effect of rinsing procedure on the aptasensor

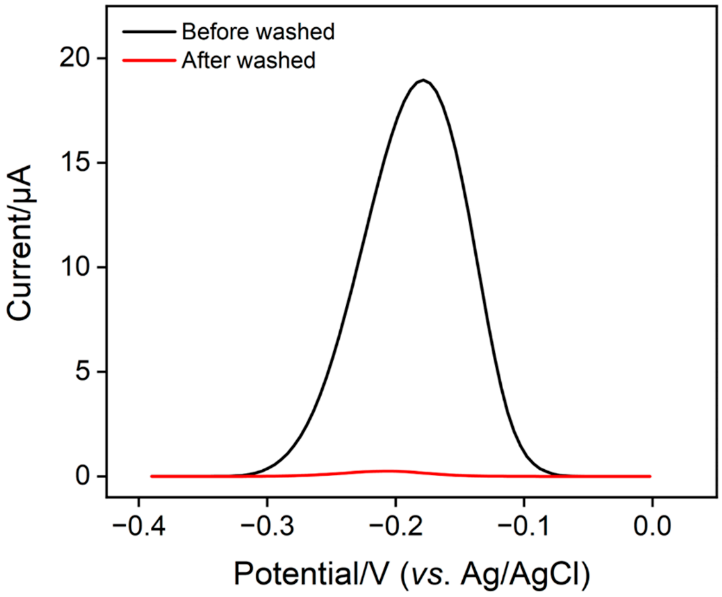

Figure S5 DPV signals of BSA/Apt/O-VMSF/ITO aptasensor in blank PBS (0.01 M pH 7.4) after incubation with 50 μL of extracted corn sample containing 10 μM $Ru(NH_3)_6^{3+}$ and 100 μM Ca^+, Na^+ and K^+ with or without being rinsed with distilled water.

S6. Anodic peak currents and their standard deviations corresponding to different concentrations

Table S1. Anodic peak currents and their standard deviations corresponding to different concentrations shown in Figure 5a.

Concentration (ng/mL)	Anodic peak current (μA)	Standard deviation (μA)
0.003	18.965	0.454
0.03	16.921	0.180
0.3	14.981	0.053
3	13.163	0.504
30	10.328	0.342
300	7.258	0.415
3000	5.663	0.265
